# The Specificity of Epizootic and Epidemiological Processes in Natural Foci of Hemorrhagic Fever with Renal Syndrome and Tick-Borne Encephalitis in Russia, as the Basis for the Prospects of Creating a Combined Vaccine for the Prevention of These Infections

**DOI:** 10.3390/v16081292

**Published:** 2024-08-13

**Authors:** Evgeniy Tkachenko, Alexandra Balkina, Dmitriy Trankvilevsky, Nadezda Kolyasnikova, Rostislav Teodorovich, Mikhail Vorovich, Yulia Popova, Svetlana Kurashova, Maria Egorova, Alla Belyakova, Petr Tkachenko, Aydar Ishmukhametov, Tamara Dzagurova

**Affiliations:** 1 Chumakov Federal Scientific Center for Research and Development of Immune-and-Biological Products of Russian Academy of Sciences, Institute of Poliomyelitis, Moscow 108819, Russiakolyasnikova_nm@chumakovs.su (N.K.);; 2 Federal Center of Hygiene and Epidemiology, Moscow 117105, Russia; trankvilevskiy@mail.ru; 3 Department of Internal Disease Propaedeutics, Sechenov First Moscow State Medical University, Moscow 119991, Russia

**Keywords:** hantavirus, hemorrhagic fever with renal syndrome, tick-borne encephalitis, reservoir hosts, natural foci, incidence rate, mortality rate

## Abstract

Hemorrhagic fever with renal syndrome (HFRS) and tick-borne encephalitis (TBE) are the most common viral diseases in Russia. HFRS is caused by six different types of hantaviruses: Hantaan, Amur, Seoul, Puumala, Kurkino, and Sochi, which are transmitted to humans through small mammals of the Muridae and Cricetidae families. TBE is caused by viruses belonging to five different phylogenetic subtypes. The similarities in the ecology of HFRS and TBE pathogens is presented here. Hantavirus-infected small mammals can transmit the virus to uninfected animals, and ticks can also transmit hantavirus to other ticks and mammals. Hantavirus transmission from ticks to humans is possible only hypothetically based on indirect data. Over the past 23 years, 164,582 cases of HFRS (4.9 per 10^5^ people) and 71,579 cases of TBE (2.5 per 10^5^ people) were registered in Russia. The mortality rate was 0.4% (668 cases) in HFRS and 1.6% deaths (1136 cases) in TBE. There were 4030 HFRS (2.5%) and 9414 TBE (13%) cases in children under 14 years old. HFRS and TBE cases were registered in 42 out of 85 Russian regions; in 18—only HFRS, in 13—only TBE, and 12 had no reported cases. The prospects of applying a combined vaccine for HFRS and TBE prevention are shown in this paper.

## 1. Introduction

Natural focal diseases are a significant public health concern in Russia among more than 64 infectious and parasitic diseases [1].

**Figure 1:**
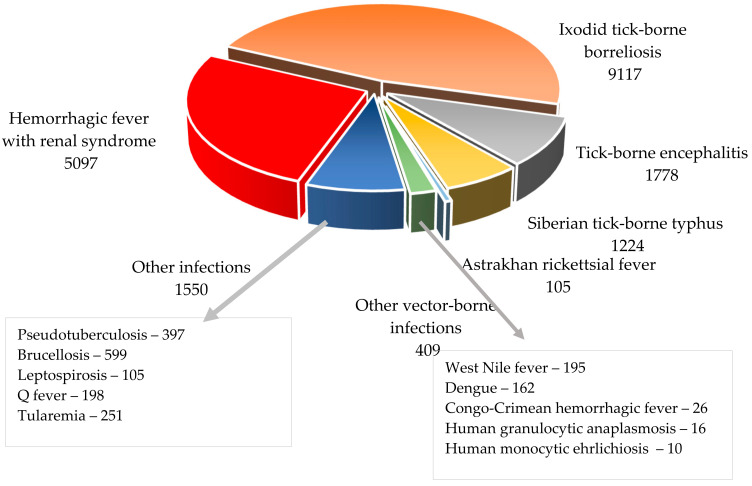
Natural focal human infections in Russia in 2023.

Thus, in 2023, more than 19,500 cases of natural focal infections were registered in Russia, including: hemorrhagic fever with renal syndrome (HFRS), tick-borne encephalitis (TBE), Crimean–Congo hemorrhagic fever, West Nile fever, Ixodid tick-borne borreliosis, Siberian tick fever, human monocytic ehrlichiosis, granulocytic anaplasmosis humans, Astrakhan rickettsial fever, brucellosis, pseudotuberculosis, tularemia, Q fever, and leptospirosis, as well as imported cases of Dengue fever and other “tropical” infections (Figure 1).

HFRS, a non-transmissible zoonosis, together with TBE, a vector-borne infection transmitted by ticks, are the most common natural focal diseases of viral etiology in Russia [2,3,4,5].

Systematic studies of HFRS and TBE began in Russia almost simultaneously, more than 85 years ago [6,7,8]. However, these natural focal infections still pose a threat to the health of residents living in endemic areas in the 21st century. The relevance of these two infections is determined by the extensive foci of their spread, high annual incidence rates in the residents, and the possibility of developing severe forms of the disease leading to permanent disability and death. HFRS has the highest incidence rate among registered zoonotic viral diseases in Russia. HFRS was included in the official registration system of the Russian Ministry of Health in 1978. From 1978 to 2023, 297,846 cases of HFRS were registered in Russia [1].

TBE ranks second after HFRS among viral natural focal diseases in Russia. It was included in the official registration system of the Russian Ministry of Health in 1944. Currently, more than 60 million people in Russia live in areas endemic for TBE, and between 2000 and 3000 cases are reported annually [3].

Due to the lack of effective etiotropic medicine for the treatment of HFRS and TBE, mass vaccination of the population in endemic areas is the only effective strategy to reduce the incidence of these infections.

TBE is a vector-borne infection that is transmitted by Ixodid ticks. A number of approved vaccines have been developed against it [9,10,11,12,13]. However, these vaccines do not offer 100% protection against TBE virus (TBEV) infection. TBE cases are registered annually among vaccinated individuals [11,14,15,16]. The incidence of TBE among vaccinated people varies depending on the endemic region and ranges from 3.7% to 23.8% of the total number of cases [17,18], and the disease more frequently occurs in a febrile form among them. However, even with a high level of vaccine-induced immunity, some vaccinated people can still develop severe disease forms with chronic sequelae and even death [19,20,21,22].

The reasons for the ineffectiveness of any medicine, including vaccines, may be due to factors associated not only with the properties of the medicine itself, but also with the host determinants and differences in the genetic and immunological presentation by the virus strains themselves (differences in the protective effect against TBEV of a heterologous subtype) [23,24].

Inactivated vaccines for HFRS prevention, which are produced in China and South Korea, are based on the Hantaan (HTNV) and Seoul (SEOV) viruses [25]. However, these vaccines are not effective against the Puumala virus (PUUV), which is responsible for more than 97% of all cases of HFRS in Russia.

Polyvalent candidate vaccine (based on PUUV, HTNV, and Sochi virus (SOCV)) successfully passed preclinical trials and, currently, a bivalent inactivated vaccine candidate against HFRS produced on the basis of PUUV and HTNV is undergoing preclinical trials in Russia [26,27].

The versatility of such a vaccine will allow its use throughout Russia as well as in Europe and Asia, since the bivalent vaccine has protective properties not only against PUUV and HTNV, but as expected also against all other currently known hantaviruses in Europe and Asia.

Due to the increased number of vaccines administered to people and the expansion of vaccination schedules in different countries, it has become necessary to simplify the use of existing vaccines. The aim can be achieved by creating combined vaccines that solve the problem of vaccinating against several diseases simultaneously. The possible advantages of combined vaccines include: reducing the antigenic load on the body, reducing the number of injections, cost savings, and less expensive materials and time needed for vaccination. Combined vaccines also reduce the risk of post-vaccination reactions and complications, as well as the number of excipients (preservatives and stabilizers) in the vaccine and, additionally, they reduce psycho-emotional stress for patients.

In recent years, a number of different combined vaccines have been developed, including DTap (diphtheria-tetanus-whooping cough), trivalent inactivated polio (IPV), measles–mumps–rubella (MMR), DTap-Haemophilus influenzae type b (Hib), and hepatitis B (Hep B). The relevance of creating and using new combined vaccines is expected to increase in the future [28].

Inactivated vaccines against HFRS [29] and TBE [30], which are produced using similar technologies, hold promise for the development and use of a combined vaccine to prevent HFRS and TBE in Russia.

This review analyzes the etiological, environmental, and epidemiological features of HFRS and TBE in Russia and substantiates the relevance and expediency of using a combined vaccine to prevent these diseases.

## 2. Etiology of HFRS and TBE

HFRS is caused by orthohantaviruses, which belong to the order *Bunyavirales*, family *Hantaviridae*, genus *Orthohantavirus.* From now on, we will refer to these viruses simply as “hantaviruses”. Hantaviruses are enveloped viruses with a negative-sense, three-segmented RNA genome that has a round shape and a diameter of approximately 90–130 nanometers [31,32].

The causative agents of HFRS in Russia are six hantavirus types, which are significantly different from each other immunologically and genetically. These viruses support their existence in nature through six different species of mammals, including mice and hamsters, from the families *Muridae* and *Cricetidae* of the order *Rodentia*. These animals serve as infection sources for humans. In the Far Eastern regions of Russia, three hantaviruses cause HFRS: the HTNV, the Amur virus (AMUV), and the SEOV. The natural reservoir hosts for these viruses are the eastern subspecies of field mice (*Apodemus agrarius mantchuricus*), East Asian mice (*Apodemus peninsulae*), and gray rats (*Rattus norvegicus*). The other three hantaviruses that cause HFRS are found in the European part of Russia. These include the PUUV, the Kurkino virus (KURV), and the SOCV. The natural reservoir hosts for these viruses are the bank vole (*Myodes glareolus*), the western subspecies of the field mouse (*Apodemus agrarius*), and the Caucasian wood mouse (*Sylvaemus ponticus*), respectively [33,34,35].

It should be noted that 97.7% of HFRS cases in Russia are caused by PUUV [36] and only 2.3% of HFRS cases are caused by the other five hantaviruses; HTNV, AMUV, SEOV, KURV, and SOCV. Thus, PUUV plays a major role in the incidence of HFRS in Russia.

Human infection with hantaviruses can occur in various ways:

1. Aerogenic (airborne dust) transmission—hantaviruses, along with aerosols containing waste products from mammals, enter the human lungs through the upper respiratory tract, where conditions for their reproduction are most favorable. From there, they can spread through the bloodstream to other organs [33,37,38,39].
2. By contact—with hantavirus-infected waste products of mammals that come into direct contact with damaged skin, mucous membranes of the eyes, nose, or mouth [40].
3. Rodent bites—infection with the saliva of animals infected with hantavirus [41,42].
4. Alimentary—with human-consumed food contaminated with hantavirus [43,44], transmission of hantavirus through dirty hands during smoking [45,46] and other activities [47]. These data suggest possible human infection through the digestive system [48].
5. Intrauterine transmission—information on monitoring pregnant women with HFRS; 10 out of 84 had stillborn children. At the same time, hantaviruses were isolated from four dead children, and antibodies to the virus were found in two surviving children, which lasted up to 3 years [49].
6. Tick bites—current evidence suggests that hantaviruses infect ticks naturally and that infected ticks can transmit hantavirus through the bite of small susceptible mammals (possibly with saliva), as well as transovarially (vertically) to their offsprings [50,51,52,53,54]. In addition, there are data on the incidence of HFRS in humans after the administration of suspensions of *Gamasoidea* and *Trombiculidae* mites [55,56,57,58,59].

Taken together, these findings challenge the current belief that rodents are the only reservoir of human pathogenic hantaviruses and that humans become infected by inhaling contaminated aerosol particles. An alternative hypothesis is that ticks, which may live on rodents or in their nesting areas, may also play a role in transmitting the virus [60]. Additional research is needed to confirm whether ticks are indeed associated with hantavirus infection and to determine if they may be the source or vector for human pathogenic hantaviruses.

In the history of HFRS research, the infection transmission through contact with HFRS patients has not been documented, particularly when caring for them in medical facilities. The causative agent of TBE is the TBEV, which belongs to the genus *Ortoflavivirus* and the family *Flaviviridae*. The TBEV is an enveloped virus with a single-stranded positive-sense RNA genome approximately 11,000 nucleotides in length and spherical virions measuring 45–50 nanometers in diameter [61].

TBEV strains belong to five different phylogenetic subtypes: Far Eastern, European, Siberian, “178–79”, and Baikal [62,63,64,65]. Additionally, the Himalayan subtype was recently identified [66]. Each TBEV subtype has a different pathogenic potential for humans, a specific habitat in which it is dominant, and is associated with a particular type of tick vector and range of vertebrate hosts. Today, the Siberian subtype is the most genetically diverse and geographically widespread, causing the entire spectrum of clinical symptoms in humans—from mild fevers to severe forms with fatal outcomes, as well as chronic forms leading to disability, and an asymptomatic course of infection [67,68]. The range of TBEV largely coincides with the ranges of its main vectors—ticks *I. persulcatus* (taiga tick) and *I. ricinus* (forest tick), and, in some regions of Siberia, also with tick *I. pavlovskyi* [69].

People are usually infected with TBEV through the bite of an infected tick (transmissible mechanism), or when the haemolymph of an infected crushed tick enters the wound. It is also possible to become infected through the consumption of unpasteurized goat’s or cattle milk that has been infected with TBEV. Dairy products made from this milk can also transmit the virus. The most common mode of transmission is through the saliva of an infected tick during blood-sucking, which is the classic route of transmission for this pathogen [11,70]. In 1962, R.B. Benda and L. Danes experimentally transmitted TBEV to laboratory mice and *Macacus cynomolgus* monkeys via the respiratory route [71]. Cases of TBE with an unknown mode of transmission have been reported [72], but all patients noted the appearance of ticks on clothes or skin, removed them from pets, and visited places located on the territory of natural foci of TBE. Thus, transmission of TBEV is possible by transmissible, contact, nutritional, and respiratory (aerogenic) routes. TBE is characterized by a strict seasonality, which is associated with increased tick activity. Humans are a random link in this ecological chain and cannot transmit the virus to other organisms (ecological dead-end).

## 3. Ecology of Hantaviruses

The causative agents of HFRS and TBE can only exist in the parasitic system formed by them, where each system participant performs a certain “function”, without which long-term existence of the parasitic system as a whole is impossible (epizootic process) [71].

To identify small mammals that can serve as hantavirus sources, animals captured in almost all landscape Russian zones were examined for the presence of hantaviruses [73,74,75,76,77,78,79,80,81,82,83,84]. Hantavirus antigen was identified in animal species belonging to six families (*Talpidae*, *Soricidae*, *Sciuridae*, *Cricetidae*, *Muridae*, *Gliridae*) of two orders (*Rodentia* and *Insectivora*). In addition to small mammals, hantavirus antigen was detected in the lungs of 13 bird species captured in the Russian Far East: gray heron (*Ardea cinerea*), striated heron (*Butorides striata*), Eurasian jay (*Garrulus glandarius*), black-faced bunting (*Emberiza spodocephala*), yellow-throated bunting (*Emberiza elegans*), coal tit (Parus ater), marsh tit (*Poecile palustris*), common pheasant (*Phasianus colchicus*), Daurian redstart (*Phoenicurus auroreus*), wood nuthatch (*Sitta europaea*), Ural owl (*Strix uralensis*), rufous turtle dove (*Streptopelia orientalis*), and hazel grouse (*Tetrastes bonasia*), while the Hantaan virus was isolated from the yellow-throated bunting (*Emberiza elegans*) [75,77].

In the European part of Russia, where the majority of HFRS cases occur, the bank vole occupies more than 90% of all infected individuals [36]. A survey of small mammals showed that in almost every landscape zone there are natural foci with varying degree of hantavirus activity. In active natural foci, almost all species of rodents and insectivores (even rare ones) are involved in the infectious process, although to a much lesser extent than the main reservoir hosts, which usually predominate in certain landscapes [81]. In addition to reservoir hosts, which are usually dominant in the territory of active natural foci, hantaviruses are occasionally detected, albeit briefly, in almost all rodents and insectivores and in some birds living in this territory (spillover). However, these species are most likely not involved in the infectious process and represent a dead-end of infection [81].

The results of experimental and field studies have allowed us to identify some common features that are inherent in hantavirus zoonotic infections [34]. These features include:

- A two-membered parasitic system “virus–warm-blooded host”, where the virus can circulate without the involvement of arthropods as vectors—these are non-transmissible zoonotic diseases.
- The presence of each hantavirus or its gene variant in only one reservoir host (species or subspecies), which is evolutionarily related and able to maintain the foci, and its inability to be replaced in this capacity by other warm-blooded animals, is the species specificity of the pathogen [82]. As a result, there are no foci of this virus outside the range of the main host, which serves as the source of infection in natural foci and is responsible for infecting people throughout the epizootic process and all phases of this virus’s range. This is why hantavirus infections differ fundamentally from other natural zoonotic diseases, which can have two or more warm-blooded animal species as their main or additional hosts and can be interchangeable in terms of carrying the pathogen both in time and space.
- The asymptomatic course of infection in the infected virus carriers which does not affect their vital activities, such as growth, reproduction, and mobility, and therefore does not impact the size or age-functional structure of populations and is known as a latent or inapparent form of infection in natural hosts.
- Lifelong persistence of the virus in the body of the reservoir host, with its reproduction being activated and released into the external environment through excreta, such as saliva, urine, and feces. This is most often seen in the first month after infection, during which time the infection is considered to be persistent with short periods of active virus carriage.

The listed features are common to various hantaviruses and undoubtedly play a significant role in the nature of infection circulation among reservoir hosts. The interaction of hantaviruses with their warm-blooded hosts, both at the organismal and population levels, and the mechanisms of pathogen transmission within the two-part parasitic system “virus-warm-blooded host” have several characteristic features. The combination of these signs determines the specific features of the modern spread and functioning of hantavirus infections natural foci and, in general, the epizootology and nature of epidemic outbreaks [81].

During all stages of the epidemic process, the pathogen circulates among populations of reservoir hosts. According to current theories, these reservoir hosts have gone through a long process of co-evolution, making these binary parasitic systems particularly stable. Species that are not related to hantavirus evolutionarily, judging by indirect evidence, can only serve as accidental hosts for the pathogen, acting as a biological dead-end for infection, such as in humans.

The nature of epizootic and epidemic processes in hantaviruses natural foci, as well as their spatial distribution, are closely related to the biology and dynamics of the populations of their main hosts. At present, it is clear that natural foci associated with different hantavirus species should be considered individually and classified according to the specific viral agent or host population.

According to one of the main principles of the concept of natural foci of hantavirus infections, hantavirus zoonosis, the area where HFRS occurs, is limited to areas where rodents carry hantaviruses that are pathogenic to humans. Hantavirus antigens detection in wild rodents directly indicates the presence of the HFRS pathogen in the area being surveyed. However, the ability to detect specific antigens is not constant, and the frequency with which animals are positive for the presence of these antigens can vary significantly over time, even within a small area. This is because of the peculiarities of the epizootic process, which can intensify or subside periodically.

## 4. Ecology of the TBE Virus

Spontaneous infection with the tick-borne encephalitis virus has been identified in 18 species of Ixodid ticks [69,85]. Among these, only two species belonging to the *Ixodes* genus are considered to be the main carriers and long-term reservoirs of the virus: *I. persulcatus* and *I. ricinus*.

Since the early 1960s, experts have identified a particularly important role for small mammals in the spread of the tick-borne encephalitis virus. These animals are crucial to the survival of the entire parasite system [86]. Intensively reproducing small mammals, which are characterized, as we know, by rapid generational change, provide a regular influx of young, non-immune individuals, and thereby create conditions for the intensive reproduction and spread of the virus [87].

The preimaginal stages of tick development, the main vectors of the virus, generally parasitize animals living in the area. At the same time, the main role of the “feeder” is assigned to the dominant small mammals in a certain period of time. The most important species as hosts of ticks throughout the forest zone are forest voles of the genus *Myodes*: the bank vole (*Myodes glareolus*), the red-backed vole (*Myodes rutilus*) and the red-gray vole (*Craseomys rufocanus*), as well as shrews of the genus *Sorex*: the common shrew (*Sorex araneus*), the medium shrew (*Sorex caecutiens*), the small shrew (*Sorex minutus*) and other shrew species. In the European part of Russia, immature ticks are fed mainly by the bank vole and the common shrew. In the Asian part of Russia, the bank vole is replaced by the red-backed vole or the red-gray vole [88]. In some European outbreaks, the European wood mouse (*Sylvaemus sylvaticus*) and the yellow-throated mouse (*Sylvaemus flavicollis*) are significant, and in the Far Eastern outbreaks—the field mouse (*Apodemus agrarius*) and the East Asian mouse (*Apodemus peninsulae*) [86,87]. In recent years, these species have predominated in catches in certain territories of Russia [89,90].

Virus strains were isolated from rodents and insectivores, which play a different role in the epizootic process, and RNA isolates of all TBEV genotypes were obtained. These included species such as: the red-backed vole (*Myodes rutilus*), the red-gray vole (*Craseomys rufocanus)*, the housekeeper vole (*Alexandromys oeconomus*), the East Asian mouse (*Apodemus peninsulae*), the narrow-skulled mouse vole (*Lasiopodomys gregalis*), the bank vole (*Myodes glareolus*), the long-tailed ground squirrel (urocitellus undulatus), the house mouse (*Mus musculus*), the European wood mouse (*Sylvaemus sylvaticus*), the field vole (*Agricola agrestis*), the field mouse (*Apodemus agrarius*), the common vole (*Microtus arvalis*), the flat-skulled vole (*Alticola strelzowi*), the big-eared vole (*Alticola macrotis*), the common shrew (*Sorex araneus*), the European mole (*Talpa europaea*), and the common squirrel (*Sciurus vulgaris*) [91].

Surprisingly, cases of isolation of the European subtype TBEV strains from the lung tissue of wild rodents in South Korea have been reported [92], which are similar to the commonly used method for hantaviruses isolation.

It should be noted that the species composition of small mammals infected with hantaviruses and TBEV is very similar (six families: *Talpidae*, *Soricidae*, *Sciuridae*, *Cricetidae*, *Muridae*, and *Gliridae* of the two orders *Insectivora* and *Rodentia*).

However, their role in the epizootic and epidemiological processes of HFRS and TBE is fundamentally different in some cases due to the species specificity of hantaviruses. *Sylvaemus uralensis*, for example, dominates the territories of the KURV foci, which cannot support the virus transmission in nature and therefore does not serve as an infection source for humans. With a low density of its reservoir host (*Apodemus agrarius*), the epizootic process in these foci fades. At the same time, for ticks that carry TBEV, the species composition of hosts does not matter.

In addition to rodents and insectivores, pre-adult stages of *I. persulcatus* and *I. ricinus* ticks also attack larger animals, such as hedgehogs (*Erinaceus europaeus*), squirrels (*Sciurus vulgaris*), and chipmunks (*Eutamias sibiricus*). These species are less important hosts for ticks compared to smaller animals, but they are still important to consider when assessing the potential for virus transmission. Medium-sized animals, such as chipmunks, can be simultaneously parasitized by a significant number of tick larvae and nymphs, which is important for assessing transmission possibilities. Moles (*Talpa europaea*) can also serve as hosts for *I. ricinus* larvae and nymphs in populated areas. Farm animals, particularly large and small livestock, play a significant role in feeding adult ticks in areas where they are regularly grazed. In many cases, livestock act as the primary source of food for adult ticks in forested areas.

Most bird species are rarely and not universally attacked by *I. persulcatus* and *I. ricinus* ticks. These ticks more regularly parasitize birds that spend a lot of time on the ground searching for food [93]. Field parasitological and experimental virological studies have shown that, among the hosts of these ticks, different species of small mammals play a major role in maintaining the circulation of the TBEV.

Ticks themselves can act not only as carriers of pathogens, but also as their amplifiers or natural reservoirs. The spread of the pathogen during an outbreak can occur partially through ticks transmitting it vertically to their offspring (transovarial transmission) or when infected and uninfected individuals feed on the same animal together (horizontal transmission), as well as through sexual contact during mating. A study on sexual transmission of TBEV in *Ixodes persulcatus* ticks found that the virus was transmitted from males to females in 50% of cases [69]. This association of TBEV with spermatogenesis suggests a significant evolutionary history between this virus and the reproductive system of male ixodid ticks.

## 5. Epidemiological Analysis of the Incidence of HFRS and TBE

Natural and social factors that influence HFRS and TBE incidence include processes occurring in natural foci, such as fluctuations in the number of vector populations and reservoir hosts. On the other hand, the scale and intensity of disease in the human population are determined by the presence and activities of people in the territory’s foci (visiting and living in endemic areas) that determine contact with sources of infections. The boundaries of natural foci for HFRS and TBE are shifting, gradually including areas that were previously considered free from these infections. At the same time, the reported geographic prevalence of human HFRS and TBE cases does not reflect the true distribution of these pathogens due to the significant frequency of remaining undetected cases [81,94,95,96], forming a “hidden” part of the epidemic process, which needs to be assessed only possible through laboratory tests (Table 1).

**Figure 2:**
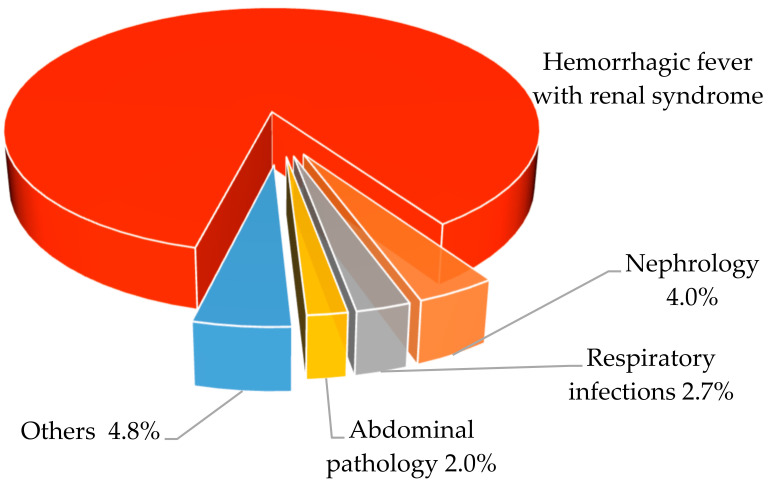
Discrepancies between specific serological and preliminary clinical diagnoses HFRS.

The detection of antibodies to hantaviruses in the blood sera of people without clinically diagnosed HFRS can be explained by a milder, even asymptomatic course of the infection. Correct and timely diagnosis of HFRS largely depends on the skills and qualifications of medical professionals. HFRS is characterized by a wide range of clinical symptoms, which greatly complicates differential diagnosis, often leading to errors (sometimes tragic) [96,97,98,99]. Serological confirmation of the HFRS clinical diagnosis in Russia as a whole exceeds 86.5%. Discrepancies between specific serological tests and preliminary clinical diagnoses are usually associated with errors in clinical diagnosis [3]. Basically, such patients were given preliminary diagnoses: respiratory infections (influenza, pneumonia, bronchitis), nephrological diseases (pyelonephritis, glomerulonephritis, renal colic, nephropathy), pathologies of the abdominal organs (acute intestinal infections, cholecystitis, pancreatitis, enterocolitis, gastroenteritis), and other infections (leptospirosis, meningococcal infection, infectious mononucleosis, fever of unknown origin) (Figure 2).

TBE differential diagnosis is carried out while also considering influenza, mumps, herpes, tuberculous meningitis, as well as with HFRS and bacterial infections transmitted by Ixodid ticks.

The use of specific laboratory tests for HFRS and TBE allows clinicians to confirm their assumptions about the possible existence of mild and asymptomatic forms of infection. The presence of mild or erased forms as well as errors in clinical diagnosis determine the level of natural immunity in the population, the value of which reflects the grade of clinical and serological diagnosis and the number of undiagnosed HFRS and TBE cases.

As a result of an epidemiological analysis of the HFRS and TBE incidence rates in Russia for the period from 2000 to 2022, 164,582 cases of HFRS were identified with an average annual incidence of 4.9 cases per 10^5^ people), as well as 71,579 cases of TBE with an average annual rate of 2.5 cases per 10^5^ people. There were 668 (0.4%) and 1136 (1.6%) deaths from HFRS and TBE, respectively; 4030 (2.5%) and 9414 (13%) children under the age of 14 years among patients with HFRS and TBE, respectively [2,3] (Table 2).

HFRS and TBE cases are distributed throughout the country unevenly. The incidence rates of these diseases vary significantly between different geographical regions [4,81]. In the European part of Russia, there were 162,044 registered HFRS cases accounting for 98.5% of the total number of cases in Russia with an average annual incidence rate of 9.7 cases per 10^5^ people. There were also 28,355 registered cases of TBE, accounting for 39.6% of the total cases in Russia, with an average annual incidence rate of 1.2 per 10^5^ people. On the other hand, in the Asian part of Russia, the incidence rates were lower. There were 2538 registered HFRS cases, representing 1.5% of the total, with an average incidence rate of 0.6 per 10^5^ people. The number of registered cases of TBE was 43,224, accounting for 60.4% of all cases, with an annual average incidence rate of 5.6 per 10^5^ people (Table 2).

The incidence of HFRS has shown a cyclical pattern over the past 23 years, with a cycle of approximately 3–4 years (Figure 2). This is likely due to the cyclic nature of the epizootic process in the foci of the PUUV [100].

**Figure 3:**
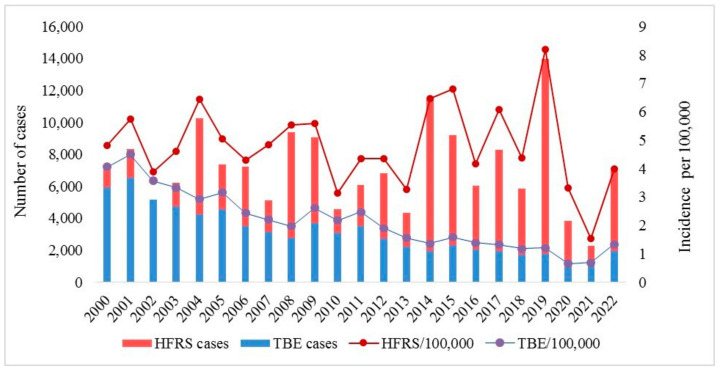
The dynamics of HFRS and TBE morbidity in Russia.

TBE also exhibits a periodic increase in incidence, with an interval of approximately 3 years. This is thought to be related to the ecology of the pathogen and its vector, the Ixodid tick [4] (Figure 3).

**Figure 4:**
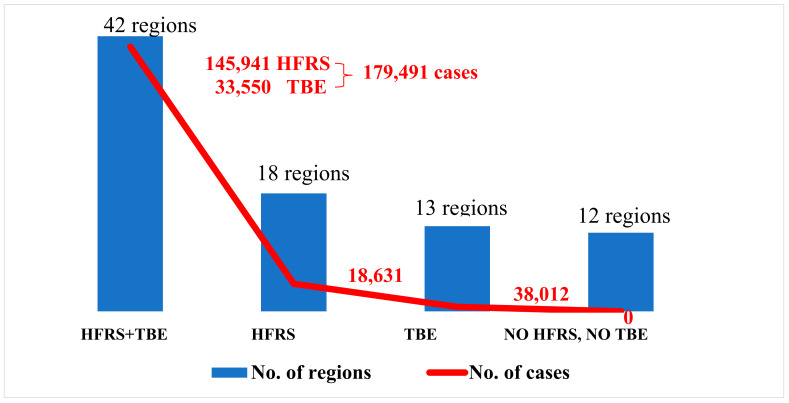
Territories with HFRS and TBE cases in Russia.

**Figure 5:**
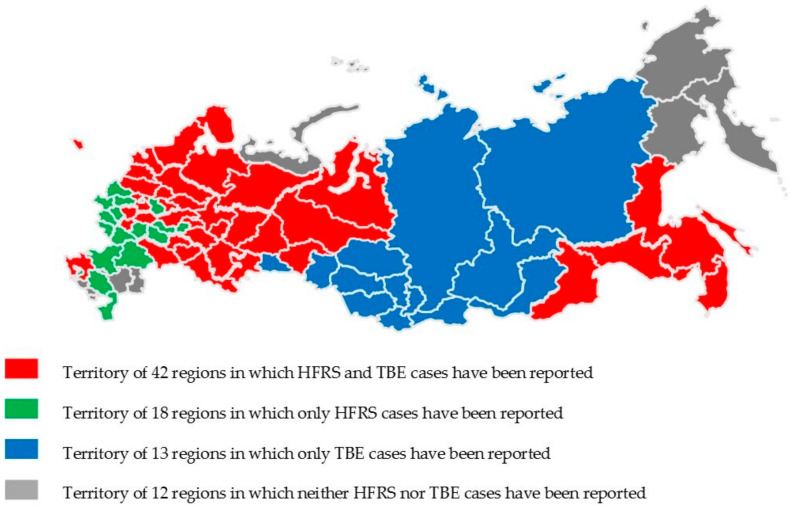
The distribution of HFRS and TBE incidence in Russia.

Russian administrative regions (85 in total) can be divided into four groups depending on the geographical territories where HFRS and TBE cases have been recorded (Figure 4 and Figure 5). The first group consists of 42 regions where both HFRS and TBE cases have been reported. The second group includes 18 regions where only HFRS cases have been identified. The third group comprises 13 regions where only TBE cases have occurred. Finally, the fourth group consists of 12 regions where neither HFRS nor TBE cases have been detected.

**Figure 6:**
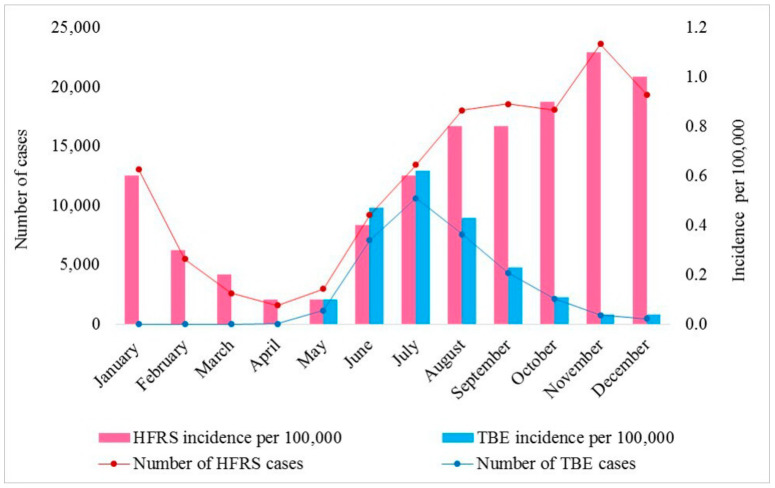
Monthly incidence in the territory of 42 regions in which HFRS and TBE cases were recorded.

The most HFRS cases were reported during the autumn-winter period in Russia. However, isolated cases were also reported throughout the year (Figure 6).

The highest TBE incidence is recorded at the end of June and the first half of July. This is due to the long incubation period of the infection (14–21 days) which is usually followed by a spring peak in tick activity [4]. The average duration of tick activity is between 60 and 65 and 120 and 140 days [69].

The main risk factor for hantavirus infection, which determines the overall HFRS incidence in Russia, is visiting forests and suburban areas and in the cold season, staying indoors in these areas. According to the study, 97.7% of HFRS cases in Russia are due to this risk factor [81].

People can become infected with TBE regardless of their occupation or economic status, and infections can occur when visiting forests or parks for recreational purposes, such as collecting mushrooms and plants [4]. More often, people in the working age group (20–50 years old) suffer from HFRS and TBE. Male patients account for 76% of HFRS cases and 65% of TBE, while female patients account for 24% of HFRS cases and 35% of TBE [81,101].

Overall, the incidence of HFRS and TBE is higher among rural residents compared to urban residents in Russia (Table 3).

## 6. Conclusions

HFRS and TBE are the most common viral diseases in Russia. Although the causative agents for these infections differ taxonomically, there is a similarity in their ecological properties, as well as a territorial overlap in the natural foci of these diseases. Based on the literature review presented in this article, several additional aspects have been revealed regarding HFRS and TBE and are listed below:

- number of small mammal species that feed ticks are infected with hantaviruses, although only reservoir hosts of viruses participate in their transmission;
- transovarial transmission of hantaviruses in ticks is possible only hypothetically and further field and experimental studies are needed for definitive proof;
- small mammals from the orders *Rodentia* and *Insectivora* retain hantaviruses and can transmit them to uninfected animals and ticks;
- ticks store hantaviruses and can transmit them to mammals and other ticks;
- transmission of TBEV from ticks to humans is generally recognized, but for hantaviruses, the causative HFRS agents, it is possible only hypothetically on the basis of indirect data;
- the true role of ticks of various taxonomic affiliations in hantaviruses natural foci and the possibility to transmit the HFRS pathogen by ticks to humans requires further field and experimental studies;
- identification of mild and erased forms of the clinical course of HFRS and TBE, as well as errors in clinical diagnosis, determine the presence of natural immunity in the population, the value of which reflects the level of clinical and serological diagnosis and the number of unaccounted patients with these infections;
- among patients with HFRS and TBE, the mortality rate is 0.4% and 1.6%, respectively; children under the age of 14 get sick in 2.5% and 13% of cases, respectively;
- HFRS and TBE incidence per 10^5^. people is higher among rural residents;
- most cases of HFRS were registered in the autumn-winter period, and TBE—at the end of June—the first half of July;
- HFRS and TBE cases are registered in 42 out of 85 administrative regions of Russia; in 18 regions—only HFRS; in 13—only TBE; in 12 regions—no clinically diagnosed HFRS or TBE cases have been identified;
- comparative epidemiological analysis of data on HFRS and TBE incidence in Russia indicates the promise of using a combined vaccine to prevent these infections.

## Figures and Tables

**Table 1:** Hantavirus antibody prevalence rate in Russia.

Area of Russia	No. of Regions Examined	No. of People Examined by IFA	No. of Positive People	% Positive People
Total for Russia	52 out of 76	65,492	2781	4.2
European Russia	38 out of 49	52,788	2504	4.7
Asian Russia	14 out of 27	12,704	277	2.2

**Table 2:** HFRS and TBE comparative incidence rates of in Russia.

Area of Russia	No. of HFRS and TBE Cases		Annual Average Morbidity Rate Per 10^5^ People		Children under 14 Years Old				Case Fatality			
	HFRS	TBE	HFRS	TBE	HFRS	%	TBE	%	HFRS	%	TBE	%
Total for Russia	164,582	71,579	4.9	2.5	4030	2.5	9414	13.0	668	0.4	1136	1.6
European part	162,044	28,355	9.7	1.2	3957	2.4	3949	14.0	572	0.4	442	1.6
Asian part	2538	43,224	0.6	5.6	73	2.9	5465	12.6	96	3.8	694	1.6
Western Siberia	300	22,206	0.4	8.0	6	2.0	2848	13.0	5	1.7	295	1.3
Eastern Siberia	9	17,765	0.1	8.6	0	-	2190	12.3	2	2.9	224	1.3
Far East	2228	3251	1.4	1.3	67	3.0	427	13.0	89	4.0	175	5.4

**Table 3:** The ratio of rural and urban residents infected with HFRS and TBE in Russia for the period from 2000 to 2022.

HFRS Cases							TBE Cases					
Area of Russia	Total	Rural Residents	Per 10^5^ People	Urban Residents	Per 10^5^ People	Ratio: Rural/Urban	Total	Rural Residents	Per 10^5^ People	Urban Residents	Per 10^5^ People	Ratio: Rural/Urban
Total for Russia	164,582	58,384	6.6	106,198	4.3	1.5	71,579	24,709	2.8	46,870	1.9	1.5
European part	162,044	5758	8.7	104,537	6.4	1.4	28,355	8827	2.1	19,528	1.2	1.8
Asian part	2538	876	0.4	1661	0.2	2	43,224	15,882	7.1	27,342	3.4	2.1
Western Siberia	300	37	0.06	263	0.05	1.2	22,206	8156	9.1	14,050	3.0	3.0
Eastern Siberia	9	2	0.04	5	0.03	1.3	17,765	6251	12.2	11,516	6.1	2.0
Far East	2229	837	1.0	1393	0.8	1.3	3251	1475	1.8	1776	1.1	1.6
